# Why Does Labor Come On?

**Published:** 1881-12

**Authors:** 


### WHY DOES LABOR COME ON?

Dr. A. Geyl, of Dordrecht, in the *Archiv fuer Gynækologie* applied the Darwinian theory to answer the questions, to which so many more or less imperfect replies have been given: Why does labor come on? and, Why does it come on at the end of the ninth month? Dr. Geyl's view is this: that it depends upon an inherited tendency to expel the child as soon as it has reached the stage of development most favorable to its separate existence, and yet permitting of its passage through the pelvis. A woman with a tendency to expel the child too soon, before it was properly viable, or to retain it too long, till it was too big to traverse the pelvis, would not of course, transmit this peculiarity to any descendant. And it is obvious that the offspring of those mothers who expelled their young at precisely the most favorable time, when the greatest degree of development compatible with safe delivery had been reached, would have a better chance of surviving than those born a little too early or too late. Dr. Geyl explains the wide differences in the duration of pregnancy by supposing that peculiarities in this direction are transmitted; *i. e.*, given a race of women with small pelves, it would be to the advantage of that race, if with the small pelvis went a tendency to the expulsion of the child before it had got very big; in the absence of that tendency the race would die out. If this were the only cause, it is plain that in the same woman pregnancy ought to always last nearly the same length of time. Dr. Geyl adduces some, but very incomplete, evidence in support of his theory. The theory, however, if true, is not an explanation. We have yet to know the mechanism by which such adaptation is effected.—*Med. and Surg. Reporter.*

